# Impact of the hypoxic microenvironment on spermatogonial stem cells in culture

**DOI:** 10.3389/fcell.2023.1293068

**Published:** 2024-01-18

**Authors:** A. S. Gille, M. Givelet, D. Pehlic, C. Lapoujade, B. Lassalle, V. Barroca, A. P. Bemelmans, D. Borderie, D. Moison, G. Livera, L. R. Gauthier, F. D. Boussin, N. Thiounn, I. Allemand, C. Peyssonnaux, J. P. Wolf, V. Barraud-Lange, L. Riou, P. Fouchet

**Affiliations:** ^1^ Université Paris Cité, CEA, Stabilité Génétique Cellules Souches et Radiations, Fontenay-aux-Roses, France; ^2^ Université Paris-Saclay, INSERM, CEA, Stabilité Génétique Cellules Souches et Radiations, Fontenay-aux-Roses, France; ^3^ Département de Génétique, Développement et Cancer. Team from Gametes to Birth, Institut Cochin, INSERM U1016, Paris, France; ^4^ Université Paris Cité, CNRS, INSERM, Institut Cochin, Paris, France; ^5^ CEA, IBFJ, Molecular Imaging Research Center (MIRCen), CNRS, Université Paris-Saclay, Fontenay-aux-Roses, France; ^6^ Université Paris Cité, Inserm, T3S, Paris, France; ^7^ Department of Biochemistry AP-HP, Cochin Hospital, Paris, France; ^8^ Université de Paris Cité, Service d’Urologie, Centre Hospitalier Georges Pompidou, Assistance Publique - Hôpitaux de Paris Centre, Paris, France; ^9^ Laboratory of Excellence GR-Ex, Paris, France

**Keywords:** spermatogonial stem cell, hypoxia, HIF, mouse model, human

## Abstract

The stem cell niche plays a crucial role in the decision to either self-renew or differentiate. Recent observations lead to the hypothesis that O_2_ supply by blood and local O_2_ tension could be key components of the testicular niche of spermatogonial stem cells (SSCs). In this study, we investigated the impact of different hypoxic conditions (3.5%, 1%, and 0.1% O_2_ tension) on murine and human SSCs in culture. We observed a deleterious effect of severe hypoxia (1% O_2_ and 0.1% O_2_) on the capacity of murine SSCs to form germ cell clusters when plated at low density. Severe effects on SSCs proliferation occur at an O_2_ tension ≤1% and hypoxia was shown to induce a slight differentiation bias under 1% and 0.1% O_2_ conditions. Exposure to hypoxia did not appear to change the mitochondrial mass and the potential of membrane of mitochondria in SSCs, but induced the generation of mitochondrial ROS at 3.5% and 1% O_2_. In 3.5% O_2_ conditions, the capacity of SSCs to form colonies was maintained at the level of 21% O_2_ at low cell density, but it was impossible to amplify and maintain stem cell number in high cell density culture. In addition, we observed that 3.5% hypoxia did not improve the maintenance and propagation of human SSCs. Finally, our data tend to show that the transcription factors HIF-1α and HIF-2α are not involved in the SSCs cell autonomous response to hypoxia.

## Introduction

In organs, the local microenvironment surrounding stem cells plays a critical role in their decision to either self-renew or differentiate. Thus, the specific local microenvironment, or niche, is critical for the regulation of tissue homeostasis. In the testis, spermatogenesis occurs in the seminiferous tubules, in which germ cells and somatic Sertoli cells are found, surrounded by myoid cells that contribute to the extracellular matrix. The interstitial space is found between these tubules. The interstitial space is notably composed of Leydig cells, blood vessels, and macrophages. Leydig cells, Sertoli cells and myoid cells were described as critical components of the niche for spermatogonial stem cells (SSCs) through the secretion of growth factors and hormones. Spermatogonia are scattered along the basal lamina of seminiferous tubules; however, undifferentiated spermatogonia, including SSCs, show a biased localization in the regions of basal lamina close to the peritubular space containing blood vessels, especially at vascular branches (Chiarini-Garcia et al., 2001; Hara et al., 2014; Yoshida, Sukeno, et Nabeshima, 2007). These observations led to the hypothesis that local O_2_ tension supplied by blood could be a key component of this niche.

In adult tissues, oxygen concentrations between 2% and 9% are thought to correspond to physiological normoxia (Brahimi-Horn et Pouysségur, 2007) and are considerably far from the ambient oxygen tensions of 21% that are usually used in culture protocols. In the testis, oxygen tensions as low as 1.5% were found inside seminiferous tubules, a relatively hypoxic environment compared with that of other tissues (Gruber et al., 2010). Hypoxia regulates diverse physiological processes and is implicated in the fate of numerous stem cells (e.g., embryonic, hematopoietic, mesenchymal, and neural stem cells) through several molecular pathways, including hypoxia-inducible transcriptions factors (HIFs), oxygen-sensitive ion channels, the environmental sensing mammalian target of rapamycin (mTOR), and the endoplasmic reticulum stress response (Mohyeldin, Garzón-Muvdi, et Quiñones-Hinojosa, 2010). The hypoxic niche is also assumed to protect stem cells from DNA damage owing to the reactive oxygen species generated by aerobic metabolism and oxidative stress. The metabolic pathway of stem cells, linked to the hypoxic niche, relies mostly on glycolysis (Mohyeldin, Garzón-Muvdi, et Quiñones-Hinojosa, 2010). Recent studies tend to show that glycolytic metabolism could be implicated in the maintenance of SSCs (Kanatsu-Shinohara et al., 2016; Helsel et al., 2017). In addition, HIF1-α is expressed in spermatogonia (Takahashi et al., 2016), and HIF1-α modulates the metabolic shift from aerobic to glycolytic metabolism (Prigione et al., 2014).

The recent preclinical assessment of SSCs therapy in nonhuman primates was of great significance for clinical translation in infertility treatment (Hermann et al., 2012). However, the efficiency of spermatogenesis recovery remains low, likely due to too few SSCs being transplanted. The amplification of SSCs *in vitro* should be a key step in increasing the stem cell number obtained from biopsies. Human SSCs can be maintained only 2–3 weeks *in vitro* (Wu et al., 2009; Medrano et al., 2016). The lack of amplification of human SSCs *in vitro* prevents the development of SSCs therapy, and new culture protocols should be developed. Stem cell culture conditions should mimic the microenvironment of the testicular niche. Thus, reducing the oxygen concentration could favor the maintenance and proliferation of SSCs *in vitro*. In this study, we investigated the impact of hypoxic conditions (3.5%, 1%, and 0.1% O_2_ tension) on murine and human SSCs in culture, as well as whether this approach could help maintain SSCs potential in culture.

## Results

### Hypoxia reduces the capacity of SSCs to form colony

**FIGURE 1:**
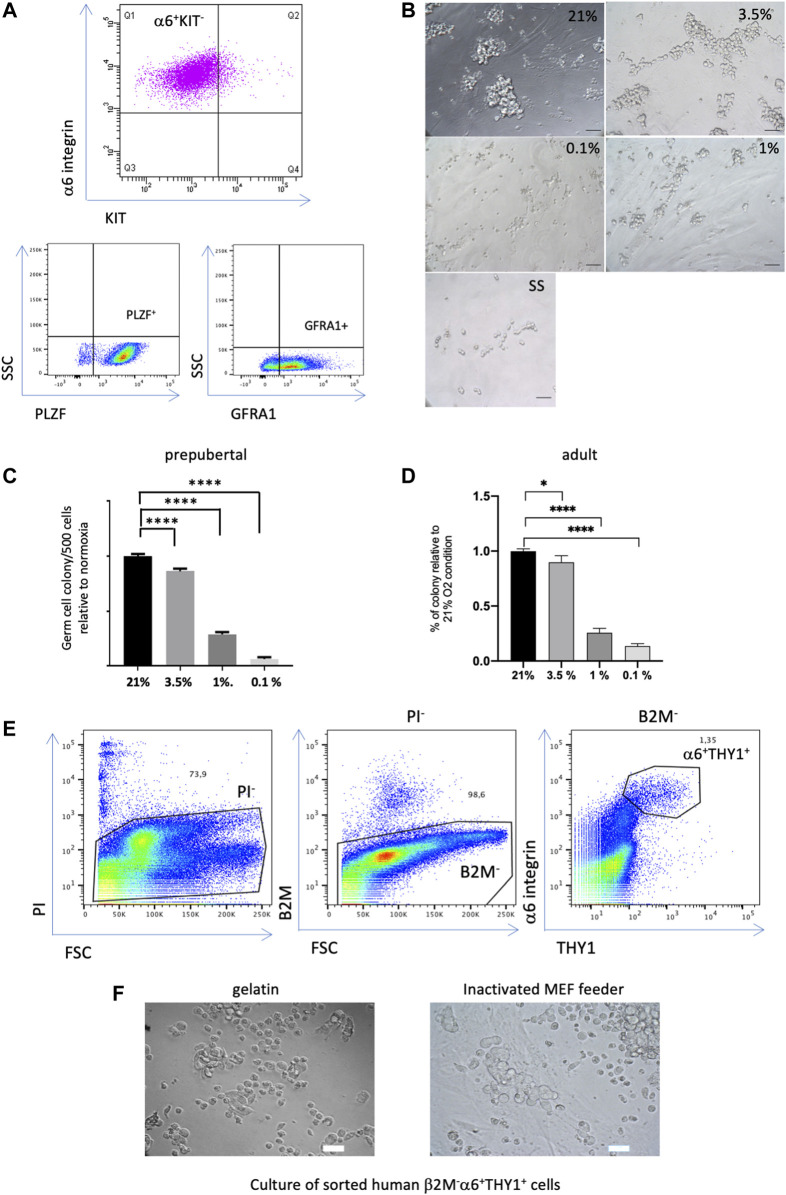
Effect of hypoxia on the colony formation capacity of SSCs **(A)** Expression profiles of undifferentiated spermatogonia markers in the culture of murine SSCs. **(B)** Morphological observation of prepubertal germ cell clusters after 7 days of culture at low density under different O_2_ tensions (21% O_2_, 3.5%, 1% and 0.1%), and under growth factor/serum-deprived conditions that induce quiescence in SSCs culture (SS condition), scale bar, 50 μm. **(C)** Analysis of the number of prepubertal germ cell clusters formed 7 days after seeding at low density (500 cells) under different conditions of O_2_ tension relative to 21% O_2_ (*n* = 30, 8 independent experiments). **(D)** Analysis of the number of adult germ cell clusters formed 7 days after seeding at low density (500 cells) under different conditions of O_2_ tension relative to 21% O_2_ (*n* = 33 for 21% and 3.5% O_2_ from 10 independent experiments, *n* = 21 for 1% O_2_ from 7 independent experiments, *n* = 9 for 0.1% O_2_ from 3 independent experiments). **(E)** Flow cytometric analysis of a human testicular cell suspension using somatic β2-Microglobulin, α6-integrin and THY1 spermatogonial markers, dead cells were discarded by propidium iodide (PI), the β2-M^-^α6^+^THY1^+^ population was identified and sorted. **(F)** Observation of the human germinal cell clusters after 2 weeks of culture of the sorted β2-M^-^α6^+^ THY1^+^ SSC population seeded on gelatin or MEF feeders, scale bar: 30 μm.

We studied the effects of O_2_ tension on murine SSCs, the best-characterized model in mammals to date. To establish these SSCs cultures, adult α6^+^c-Kit^-^β2M^−^ and prepubertal EpCAM^+^ β2M^−^ germinal cell fractions were flow sorted and seeded on mitomycin-C-treated MEFs in low-serum medium in the presence of GDNF, FGF2 and GFRA1 at 21% O_2_ as previously described (Barroca et al., 2009; Corbineau et al., 2017; Dura et al., 2022; Givelet et al., 2022). The culture of murine SSCs is relatively heterogeneous, containing self-renewing undifferentiated spermatogonia and their immediate differentiating progeny. Germ cells in SSCs culture homogenously express α-6 integrin and PLZF and are negative for the receptor c-kit, corresponding to the phenotype of the undifferentiated spermatogonial population (Figure 1A). In addition, SSCs in culture express the GDNF receptor GFRA1, which is a marker of the SSCs population with selfrenewing potential (Hara et al., 2014). Nevertheless, the *in vitro* germinal cluster formation assay provides a reliable technique to assess the number and functionality of SSCs, i.e., their capacity to form germ cell clusters starting from very low cell density (Yeh, Zhang, et Nagano, 2007). Thus, this test was used to study the effects of hypoxia on established cultures of SSCs (>1 month of culture), obtained from pups and adults, at various O_2_ tensions, namely, 21%, 3.5%, 1%, and 0.1%.

When SSCs derived from pups were seeded at low density for colony formation assays, morphological observations after 7 days of culture under the different O_2_ tensions clearly showed that germ cell clusters differed in appearance. Specifically, the 21% and 3.5% O_2_ cultures presented large clusters with typical grape-like cell morphology, while the 1% O_2_ and 0.1% O_2_ cultures displayed small cell clumps or single cells (Figure 1B). The germ cell colonies were counted, showing that severe hypoxia (1% to 0.1% O_2_) drastically decreased the colony formation potential of SSCs (Figure 1C). Hypoxia at 3.5% O_2_ only mildly modified the number of germ cell clusters compared with 21% O_2_ conditions. Germ cell colony formation assays were also performed on SSCs derived from adult mice. A similar pattern of effect on the capacity of adult SSCs to form germ cell clusters was observed in hypoxic conditions (Figure 1D). In addition, we investigated whether human SSC culture at 3.5% O_2_ could improve the *in vitro* maintenance and propagation of human SSCs. Indeed, prepubertal and adult human SSCs show only a limited potential of maintenance *in vitro* up to 2–3 weeks of culture (Wu et al., 2009; Medrano et al., 2016; Givelet et al., 2022). Thus, we tested the ability of the spermatogonial population expressing the markers α-6 integrin and THY1, and negative for the β-2 microglobulin, which we identified as highly enriched in SSCs, to form *in vitro* germinal clusters in medium supplemented with GDNF and FGF2 factors (Givelet et al., 2022). We previously demonstrated that PIWIL4, C19orf84, TSPAN33, PLPPR3, FGFR3, and UTF1 primitive spermatogonial markers were expressed in this population, and that it did not contain somatic cells (Givelet et al., 2022). The human β-2M^−^α-6^+^THY1^+^ population was sorted (Figure 1E) and seeded on gelatin-coated plates or an inactivated MEF feeder, as both were proficient at generating human germinal clusters at 21% O_2_ (Givelet et al., 2022). Cluster formation was clearly observed up to 2 weeks (Figure 1F) but ultimately vanished under both 21% O_2_ and 3.5% O_2_ hypoxic conditions. Thus, hypoxia does not appear to be a critical key factor to favor the long-term maintenance of human SSCs under the present culture conditions we used.

### The proliferation of SSCs is strongly affected by severe hypoxia in culture

**FIGURE 2:**
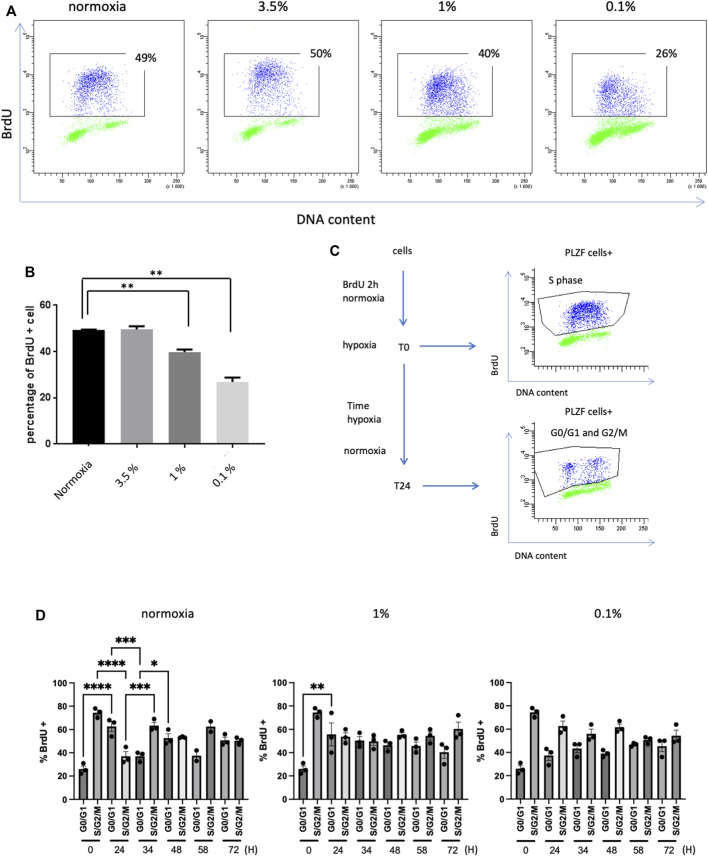
Proliferation of SSCs in hypoxia **(A)** BrdU expression in PLZF^+^ cells from prepubertal SSCs cultures analyzed by flow cytometry. **(B)** Analysis of the percentage of PLZF^+^ BrdU^+^ cells from prepubertal SSC cultures after 12 h under hypoxic conditions and 2 h of BrdU pulses (*n* = 3, Student T test). **(C)** Study of the cell cycle by BrdU pulse-chase: cultures of SSCs were pulse-labeled with BrdU for 2 h under 21% O_2_. After removal of BrdU, cells were allowed to continue cycling for a chase period of up to 72 h under 21% and 1% and 0.1% O_2_ conditions. At T0, the PLZF^+^ cells were in S phase. After 24 h, the PLZF^+^ cells had transited through the G2/M phase and returned to the G0/G1 phase. **(D)** Characterization of the cell cycle by analysis of the mean of the percentage of the G0/G1 population and S/G2/M population in the pulse chase (*n* = 3 except for 21% O_2_ at *t* = 58h, *n* = 2).

As hypoxia is a well-known modulator of cell proliferation, we evaluated the cell cycle progression and the proliferation of murine SSCs through the measurement of cells entering S phase by performing the BrdU cell proliferation assay and analysis by dual parameter flow cytometry. After a 2-h pulse of BrdU, approximately 50% of the PLZF-positive cells in SSCs culture entered S phase in 21% O_2_ conditions (Figures 2A, B). Culture under the 3.5% O_2_ condition displayed a similar proliferation rate as that under 21% O_2_. A progressive decrease in the percentage of cells in S phase was observed when SSCs were cultured in 1% O_2_ and 0.1% O_2_, indicating a deleterious effect of severe hypoxia on G_1_/S transit and progression to S phase.

To better characterize these proliferation defects at 1% O_2_ and 0.1% O_2_, we conducted a BrdU pulse-chase experiment. Cultures of SSCs were pulse-labeled with BrdU for 2 h under 21% O_2_ condition. Then, after the removal of BrdU, the cells were allowed to continue cycling for a chase period up to 72 h under 21% O_2_, 1% O_2_ and 0.1% O_2_ conditions. Cells were harvested at different times during this period (Figure 2C). BrdU-labeled PLZF-positive cells from 21% O_2_ condition were observed to exit S phase and return to G_0_/G_1_ after 24 h, while a proportion of new, unlabeled PLZF-positive cells entered S phase (Figures 2C, D). After 24 h of culture at 0.1% O_2_, the number of BrdU-labeled cells that remained in S/G_2_/M phase increased two-fold compared with that under 21% O_2_ conditions, indicating that significant hypoxia partly blocked or strongly slowed cell cycle progression, preventing reentry of labeled PLZF-positive cells into G_0_/G_1_ phase (Figure 2D). PLZF-positive cells returned to their initial cell cycle state (G_0_/G_1_ phase or S/G_2_/M) periodically every 24–34 h during the analysis period. In contrast, PLZF-positive cells did not oscillate between the G_0_/G_1_ and S/G_2_/M phases during the chase period under 1% O_2_ and 0.1% O_2_ conditions; remarkably, the majority of cells remained in the S/G_2_/M phases during this time. Together, these results show that severe hypoxia tends to arrest the cell cycle of SSCs, slowing down the G_1_/S transit and the S/G_2_/M cell cycle progression.

### Severe hypoxia favors a low exit of SSCs in culture toward quiescence

**FIGURE 3:**
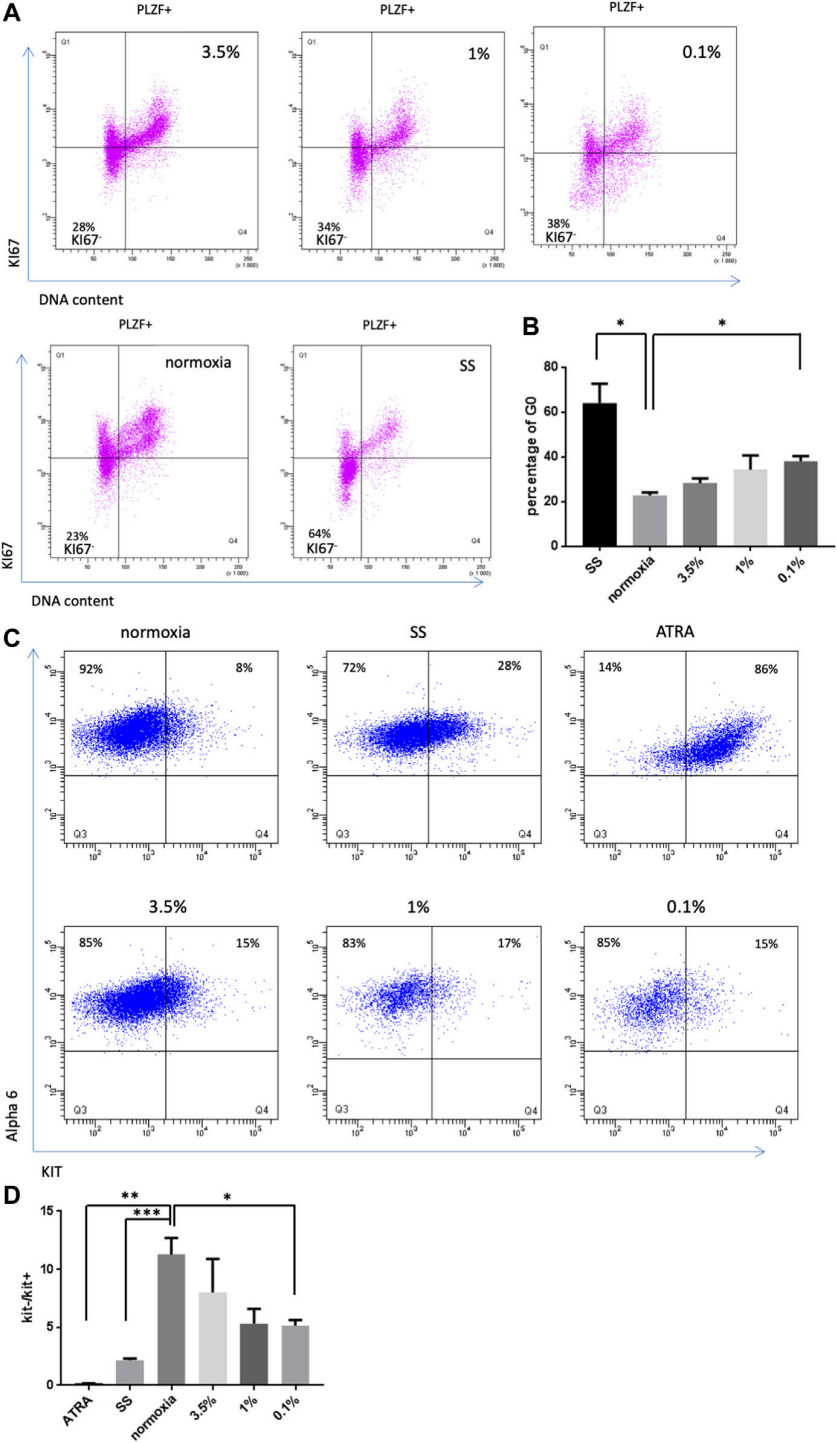
Effect of hypoxia on SSC quiescence and differentiation **(A)** KI67 labeling in PLZF^+^ SSCs population under hypoxic conditions (3.5%, 1% and 0.1%), 21% O_2_ and growth factor/serum-deprived conditions (SS). **(B)** Analysis of the percentage of G0 cells (KI67^neg^ cells) after 4 days under hypoxic conditions or growth factor/serum-deprived conditions (SS) (*n* = 3). **(C)** Observation of the differentiation of SSCs by KIT receptor expression in PLZF^+^ cells under hypoxic, differentiation (ATRA) or growth factor/serum-deprived conditions after 4 days of culture. **(D)** Analysis of the ratio of the number of KIT^−^ cells to KIT^+^ cells (*n* = 3).

We previously observed that germ cell clusters in culture showed similar morphological aspects under severe hypoxia conditions (0.1% and 1% O_2_) and growth factor/serum-deprived conditions (Figure 1B). Culture under growth factor/serum-deprived conditions induced quiescence in SSCs culture, as demonstrated by the increased number of PLZF-positive cells showing no expression of the cell cycle marker Ki-67 (Figure 3A). Thus, we analyzed whether hypoxia favors the exit from the cell cycle of SSCs and their entry into quiescence. The expression of Ki-67 was investigated in PLZF-positive cells in culture under 21%, 3.5%, 1%, and 0.1% conditions (Figure 3A), and the percentage of quiescent cells (Ki-67^-^ cells/G0) was calculated (Figure 3B). An increase in the number of cells exiting the cell cycle was observed in the 0.1% O_2_ culture condition, but the increase remained modest compared with that under growth factor/serum-deprived conditions (Figure 3B). Proliferative defects and loss of colony-forming activity appear to not be linked to a cell cycle exit under hypoxia.

### Differentiation of SSCs under severe hypoxic conditions

Next, we investigated whether hypoxia could lead to a differentiation bias of SSCs in culture. KIT receptor expression is a key marker of differentiation, defining the transition from undifferentiated spermatogonia to differentiating spermatogonia. Thus, we analyzed whether hypoxia induced KIT expression in SSCs culture under hypoxic conditions. All-trans retinoic acid (AtRA, a retinoic acid component) was previously reported to stimulate the expression of KIT and the differentiation of germ cell clumps in SSCs culture (Yang et al., 2013). AtRA-stimulated SSCs culture was used as a positive control, and we found by flow cytometric analysis that 86% of α-6^+^ undifferentiated spermatogonia became KIT^+^ following this treatment (Figure 3C). A KIT expression study was performed in SSCs culture under the different hypoxic conditions, and a ratio of the number of KIT^−^ cells to KIT^+^ cells was calculated (Figure 3D), revealing a trend toward a slight bias of differentiation under 1% and 0.1% O_2_ conditions.

### Effects of hypoxia on spermatogonial mitochondria

**FIGURE 4:**
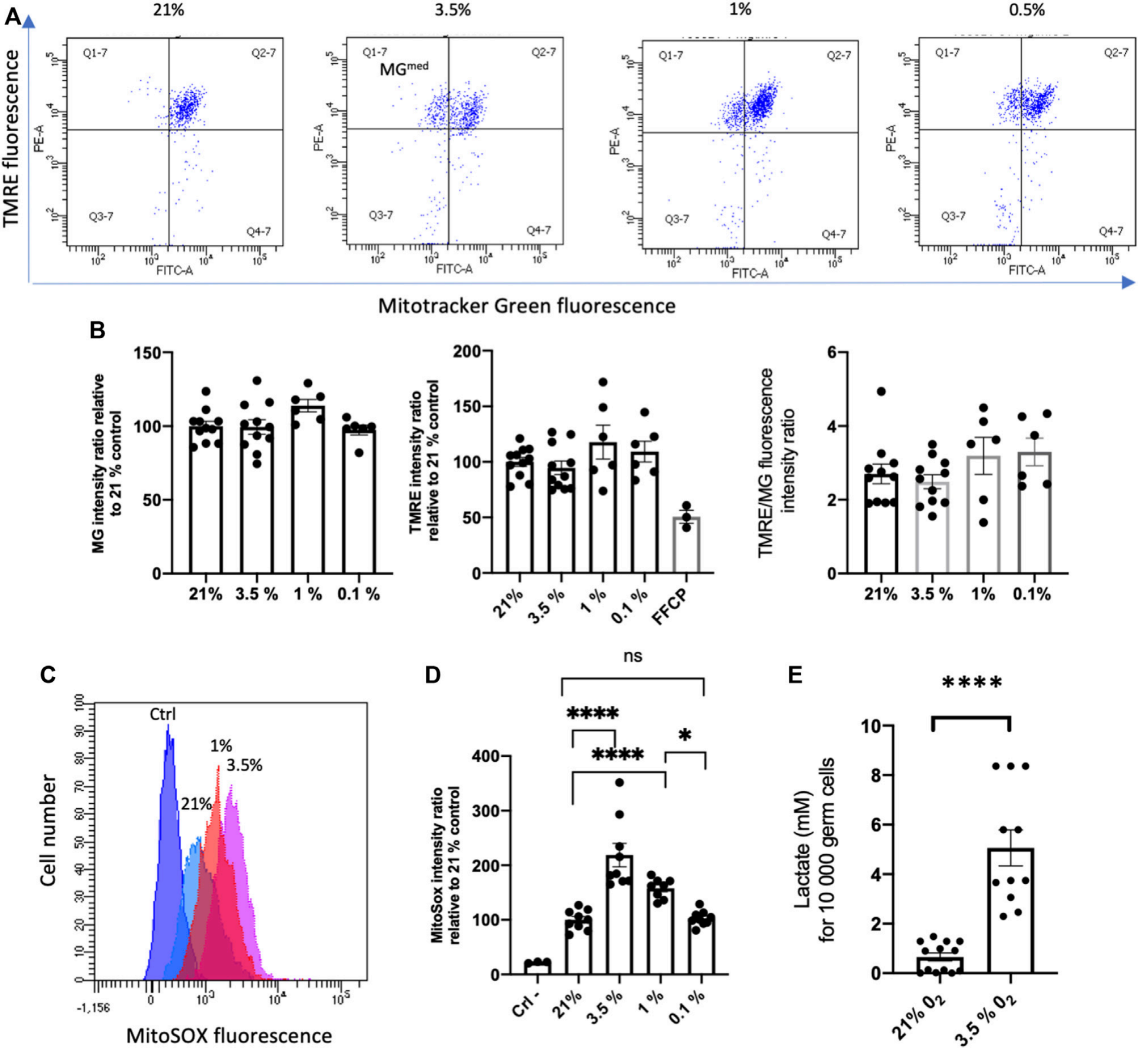
Effects of hypoxia on mitochondrial mass and potential of membrane **(A)** Flow cytometric analysis of Mito Tracker Green (MG) and Tetramethylrhodamine ethyl ester (TMRE) fluorescence in SSCs cultured during 24 in hypoxia. **(B)** Mean of MG fluorescence, TMRE fluorescence and ratio of TMRE/MG fluorescence of SSCs in the different hypoxic conditions. The specificity of the TMRE signal was controlled by its inhibition in presence of the mitochondrial oxidative phosphorylation uncoupler FCCP. TMRE and MG experiments: 21% and 3.5% O_2_ (*n* = 11, 4 independent experiments), 1% and 0.1% O_2_ (*n* = 6, 2 independent experiments). Difference between 21% group and all groups are ns for MG fluorescence, for TMRE fluorescence except for comparison with 21%/FCCP (*p* = 0.006), and for TMRE/MG ratio. **(C)** Overlay histograms of MitoSOX fluorescence in SSCs cultured during 24 h in hypoxia (hypoxic conditions are indicated). **(D)** Mean of MitoSOX fluorescence of SSCs in the different hypoxic conditions (ctrl, *n* = 3; 21%, 3.5%, 0.1%, *n* = 9; 1%, *n* = 8). **(E)** Lactate production by SSCs cultivated 4 days on MEFS in 3.5% O_2_ (*n* = 11) condition compared to 21% O_2_ (*n* = 13). A similar increase of lactate was observed at 3.5% O_2_ when maintained 4 days on laminin (data not shown).

ATP, source of energy, is mainly produced in mitochondria through oxidative phosphorylation, and ROS are by-products of the mitochondrial respiration (Finkel, 2012). As major players into oxygen sensing pathways, the mitochondria could be involved in the response of SSCs to hypoxia through modulation of oxidative phosphorylation and ROS production. Mitochondria are key actors in the regulation of stem cell quiescence and the switch to a metabolically active state (Zhang et Sadek, 2014). Mitochondrial dysfunction could indeed lead to detrimental effect on SSCs. Tetramethyl rhodamine ethyl ester (TMRE) a cationic fluorophore, which accumulates electrophoretically in polarized mitochondria was used to assess the mitochondrial membrane potential ΔΨ_m_. We used also the MitoTracker Green (MG), a mitochondrial selective probe that binds to mitochondrial proteins to evaluate the mitochondrial mass. We did not observe any significant changes in the mean MG signal of the SSCs population according to hypoxic conditions, but we noted a subpopulation with a lower MG fluorescence intensity (MG^med^) appearing in normal and hypoxic culture (Figures 4A, B). The specificity of the TMRE signal in SSCs was controlled by its inhibition in presence of the mitochondrial oxidative phosphorylation uncoupler carbonyl cyanide 4-(trifluoromethoxy)phenylhydrazone (FCCP) (Figure 4B). TMRE signal and TMRE/MG ratio of SSCs in hypoxia were roughly similar to 21% O_2_ condition, but we did not observe a loss of ΔΨ_m_ associated with the metabolic shift from OXPHOS to glycolysis. These results were unanticipated as hypoxia is often associated with decreased oxidative phosphorylation (Wheaton et Chandel, 2011), a lower activity of oxidative phosphorylation in SSCs was expected when cultured in hypoxic condition even at 3.5% O_2_. Superoxide generated by mitochondria as a consequence of oxidative phosphorylation is the major cellular source of ROS. We quantified superoxide levels in the mitochondria using MitoSOX Red, a selective fluorogenic dye that reacts specifically with mitochondrial superoxide. Paradoxically, we observed an increase of MitoSOX Red fluorescence in 3.5% and 1% O_2_ conditions compared with 21% O_2_, and levels of mitochondrial ROS were at similar levels in extreme hypoxia at 0.1% O_2_ and at 21% O_2_ (Figures 4C, D). Hence, mitochondrial metabolism and OXPHOS, reflected by the maintenance of the ΔΨ_m_ and the mitochondrial mass, seems to be active in spermatogonia in 3.5% and 1% O_2_ hypoxic conditions. However, an increase of lactate production in SSCs cultivated 5 days at 3.5% O_2_ was observed, reflecting a change in the SSCs metabolism and suggesting a potential shift to glycolysis in hypoxic condition (Figure 4E).

### Detrimental effect of 3.5% O_2_ hypoxic condition at high cell density culture of SSCs

**FIGURE 5:**
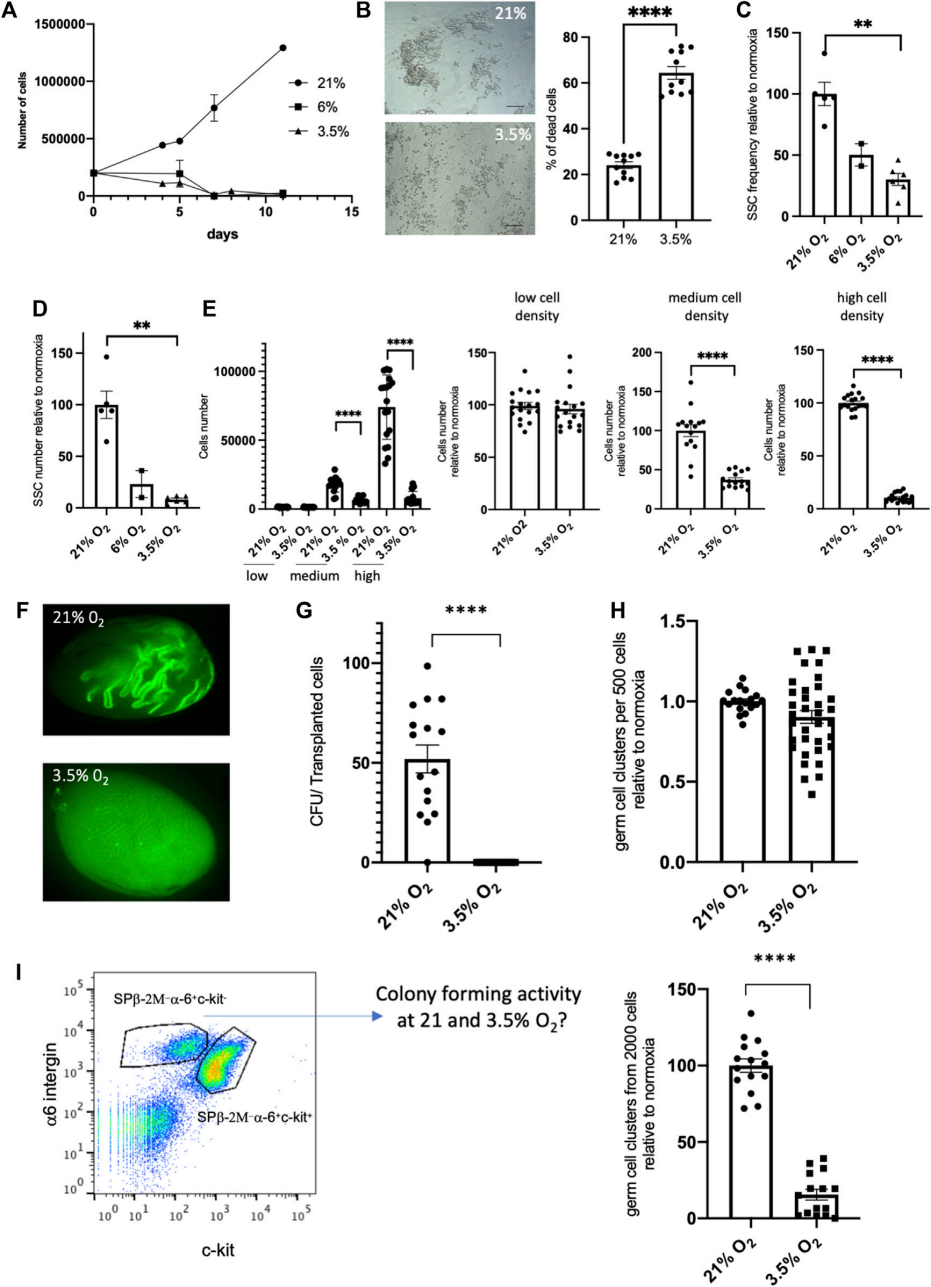
Effect of cell density on the potential of culture of SSCs in 3.5% O_2_ condition **(A)** Growth of SSCs at high cell density (30,000 cells/96 well) on MEFs feeder in 21%, 6% and 3.5% O_2_ conditions. **(B)** Cell death in culture of SSCs in 3.5% O_2_ condition: representative transmitted light images (scale bar: 100 μm) and quantification of cell death after 5 days of culture (*n* = 11, 3 independent experiments). **(C)** SSCs frequency based on their *in vitro* cell cluster forming ability after 7 days cell culture at high cell density in 21%, 6% and 3.5% O_2_ conditions. **(D)** SSCs number after 7 days cell culture at high cell density in 21%, 6% and 3.5% O_2_ conditions (frequency of SSCs x number of total germ cells in culture). **(E)** SSCs number after 7 days cell culture at low, medium and high cell density in 21% and 3.5% O_2_ conditions. Data are displayed as absolute cell numbers in each cell density condition (left graph) and as cell numbers relative to this at 21% O_2_ in each cell density condition (3 right graphs). **(F)** Fluorescent images representative of recipient testis showing EGFP-donor derived spermatogenesis in seminiferous tubules transplanted with EGFP SSCs after 7 days culture in high cell density in 21% and 3.5% O_2_ conditions. **(G)** Number of colonies generated per 10^5^ transplanted cells cultured in high cell density in 21% and 3.5% O_2_ conditions. **(H)** *In vitro* cell cluster forming ability of SSCs after hypoxic/21% O_2_ exposure. SSCs were cultured for 7 days under hypoxic conditions and then reseeded in 21% O_2_ conditions during 7 days. **(I)** *In vitro* cell cluster forming ability of sorted β2M^−^α−6^+^c-kit^−^ undifferentiated spermatogonia from EGFP mice testicular cell suspension seeded in hypoxic 3.5% O2 condition.

In an attempt to improve the amplification and maintenance of SSCs *in vitro*, we aimed to culture SSCs at long term in high cell density conditions (30,0000 cells/96 well) under 3.5% O_2_ hypoxic environment. Unexpectedly, we observed rapidly a dramatic decrease of the cell number in the SSCs culture (Figure 5A), with an increase of cell death (Figure 5B) compared to control at 21% O_2_ culture in which cell number amplified. Likewise, this decay was observed in culture at 6% O_2_. At the endpoint of SSCs culture under 3.5% O_2_ hypoxic, cells were harvested and tested for their *in vitro* cell cluster forming ability. This test confirmed that the SSCs functionality was impaired compared to 21% culture, showing a severe decrease of the frequency of cells having the ability to form colonies (Figure 5C), and of the SSCs numbers in the culture (Figure 5D). The seeding at different cell densities were tested for culture at 3.5% O_2_, namely at low cell (1,000), medium (10,000) and high (30,000) cell density per 96 wells. These experiments confirmed the positive impact that high cell density has in increasing SSCs numbers at 21% O_2_ and highlighted that the cell density is a critical parameter for the success of SSCs culture at 3.5% O_2_ compared to 21% O_2_ condition, and that only cultures at low cell density were efficient (Figure 5E). Finally, we checked using transplantation assay the effect of a 7-days culture at high cell density under 3.5% O_2_ on SSCs. This confirmed that SSCs number was significantly reduced after a 7-days culture (Figures 5F, G) When SSCs were cultured for 7 days under hypoxic and 21% O_2_ conditions and then reseeded in 21% O_2_ conditions during 7 days (hypoxic/21% O_2_ sequence), we observed that the detrimental effect of hypoxia culture on SSCs was reversible as the frequency of cells with *in vitro* cell cluster forming ability was similar in SSCs populations derived from hypoxic condition or control culture at 21% O_2_ (Figure 5H).

As we used SSCs from culture established at 21% O_2_ in our experiments, we wondered whether the detrimental effects of hypoxia on SSCs that we observed could be linked to an adaptation of the SSCs to 21% O_2_ condition, before their transfer to hypoxia. For this purpose, we sorted β2M^−^α−6^+^c-kit^−^ undifferentiated spermatogonia from testicular cell suspension of EGFP mice, and tested their ability to form *in vitro* cell cluster and to establish SSC culture in hypoxic 3.5% O_2_ condition. *In vitro* cell cluster forming ability of sorted SSCs was severely impaired at 3.5% O_2_ compared to 21% O_2_ (Figure 5I), and we did not succeed in establishing SSCs culture under 3.5% O_2_ tension. Although we cannot rule out that this observation results from a higher stress for cells owing to the combined effects of cell sorting and hypoxic culture condition, a detrimental effect of hypoxia to SSCs in culture seems not to be due to adaptation of SSCs to 21% O_2_ condition during the phase of establishment of the SSCs culture before transfer to hypoxia.

**FIGURE 6:**
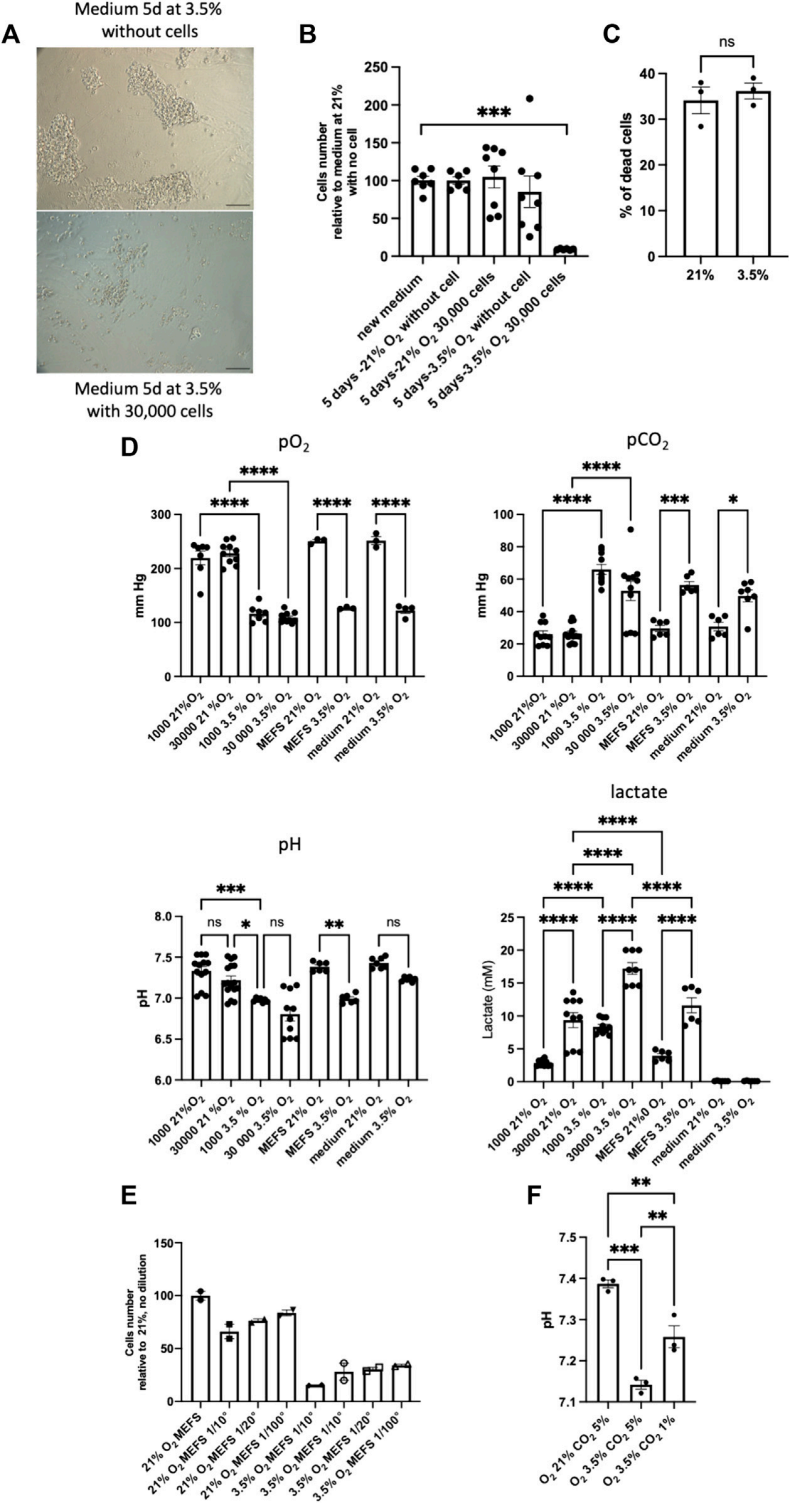
Detrimental effects of the medium of high cell density culture on MEFS at 3.5% O_2_ **(A)** Transmitted light images representative of the effects of medium harvested after 5 days of high density cell culture at 3.5% O_2_ and used as conditioned medium on new SSCs culture in 21% O_2_ condition, scale bar: 100 μm. **(B)** Cell number after 7-days culture on MEFS feeder in presence of medium conditioned during 5 days at various cell density (0 and 30,000 cells) and tension of O_2_ (21% and 3.5% O_2_), 21% new medium (*n* = 7), 21% 5 days without cells (*n* = 6), 21% 5 days high cell density (*n* = 8), 3.5% 5 days without cells (*n* = 8), 3.5% 5 days high cell density (*n* = 6). **(C)** Cell death in inactivated MEFs feeders grown under 21% and 3.5% O_2_ culture conditions (*n* = 3). **(D)** pO_2_, pCO_2_, pH and lactate dosage in media after 4-days culture on MEFs feeder at various SSCs density (0, 1000 and 30,000 cells) and tension of O_2_ (21% and 3.5% O_2_), in media on MEFs feeder only (MEFs), and in media without MEFs and SSCs (medium). pO_2_: 1000 cells, 21% and 3.5% O_2,_ *n* = 7; 30,000 cells, 21% and 3.5% O_2,_ *n* = 10; MEFs, 21% and 3.5% O_2_; *n* = 3; medium, 21% (*n* = 3) and 3.5% (*n* = 4) O_2_. pCO_2_: 1000 cells, 21% and 3.5% O_2,_ *n* = 10; 30,000, 21% (*n* = 13) and 3.5% (*n* = 11) O_2_; MEFs, 21% and 3.5% O_2_; *n* = 6; medium, 21% (*n* = 6) and 3.5% (*n* = 7) O_2_. pH: 1000 cells, 21% (*n* = 13) and 3.5% (*n* = 7) O_2,_; 30,000 cells, 21% (*n* = 16) and 3.5% (*n* = 10) O_2_; MEFs, 21% and 3.5% O_2_; *n* = 6; medium, 21% (*n* = 7) and 3.5% O_2_ (*n* = 6). Lactate: 1000 cells, 21% and 3.5% O_2_ (*n* = 10); 30,000 cells, 21% (*n* = 10) and 3.5% (*n* = 8) O_2_; MEFS, 21% and 3.5% O_2_; *n* = 6; medium, 21% and 3.5% O_2_ (*n* = 6). **(E)** Number of cells generated after 7 days cell culture at high cell density on various MEFs feeder cell density in 21% and 3.5% O_2_ conditions (*n* = 2). **(F)** pH of the medium when CO_2_ tensions of 1% and 3.5% are applied to the culture.

As we observed the reversibility of the effect of hypoxia on SSCs when exposed cells were once again cultured at 21% O_2_, we tested the effects of the medium harvested after 5 days in high cell density culture at 3.5% O_2_ on new SSCs culture in 21% O_2_ condition (Figures 6A, B). We observed clearly the negative impact of the medium from 5 days high density cell culture at 3.5% O_2_, meaning that some metabolic components/physicochemical parameters of the medium have adverse effects on SSCs. In line with this, we noted that changes of medium after 4 days of culture slightly improved SSCs maintenance at 3.5% (data not shown). At 3.5% O_2_, the negative effects could result from a greater consumption of a key growth/survival factor or a greater production of a toxic metabolite. Of note, we observed that inactivated MEFs feeders displayed similar levels of dying cells under 21% and 3.5% O_2_ culture conditions (Figure 6C). To go further, we analysed O_2_, CO_2_, lactate concentrations and pH of the medium after 21% and 3.5% culture at low (1,000) and high (30,000) cell density (Figure 6D). We observed that O_2_ tension in the medium did not vary according to the cell density at 21% or 3.5% O_2_, meaning that O_2_ supply is not limiting at high cell density. A strong increase of the CO_2_ content in the medium of all the 3.5% O_2_ conditions was noted compared to 21% O_2_, although 5% CO_2_ supply was the same whatever the conditions of culture. Remarkably, we observed a decrease of the pH of the medium in all the conditions at 3.5% O_2_, compared to 21% O_2_ condition, and the lactate concentration was the highest in high cell density culture at 3.5% O_2_. The 3.5% O_2_ culture at high cell density displayed the lower pH of all conditions tested, suggesting that this low pH could be the key detrimental parameter in the culture at high cell density at 3.5% O_2_. This low pH could be linked to the increase of lactate we observed and/or the increase of CO_2_ content in the medium. As we had observed that MEFs feeder contributed to lactate production at 3.5% O_2_, we tried to culture SSCs with lower density of feeders, a positive effect on SSCs culture at high density was noted, but the effect was relatively modest (Figure 6E), and did not allow to perform amplification of cells at 3.5% O_2_ at high cell density (data not shown). We also tried to restore the pH of the culture by modulating the CO_2_ tension applied in the incubator, we noted that pH was partially restored to the level of 21% O_2_ condition (Figure 6F), but we did not observe any positive effect on the culture.

### HIF-2α and HIF-1α do not play a role in the self-autonomous response of SSCs to hypoxia

**FIGURE 7:**
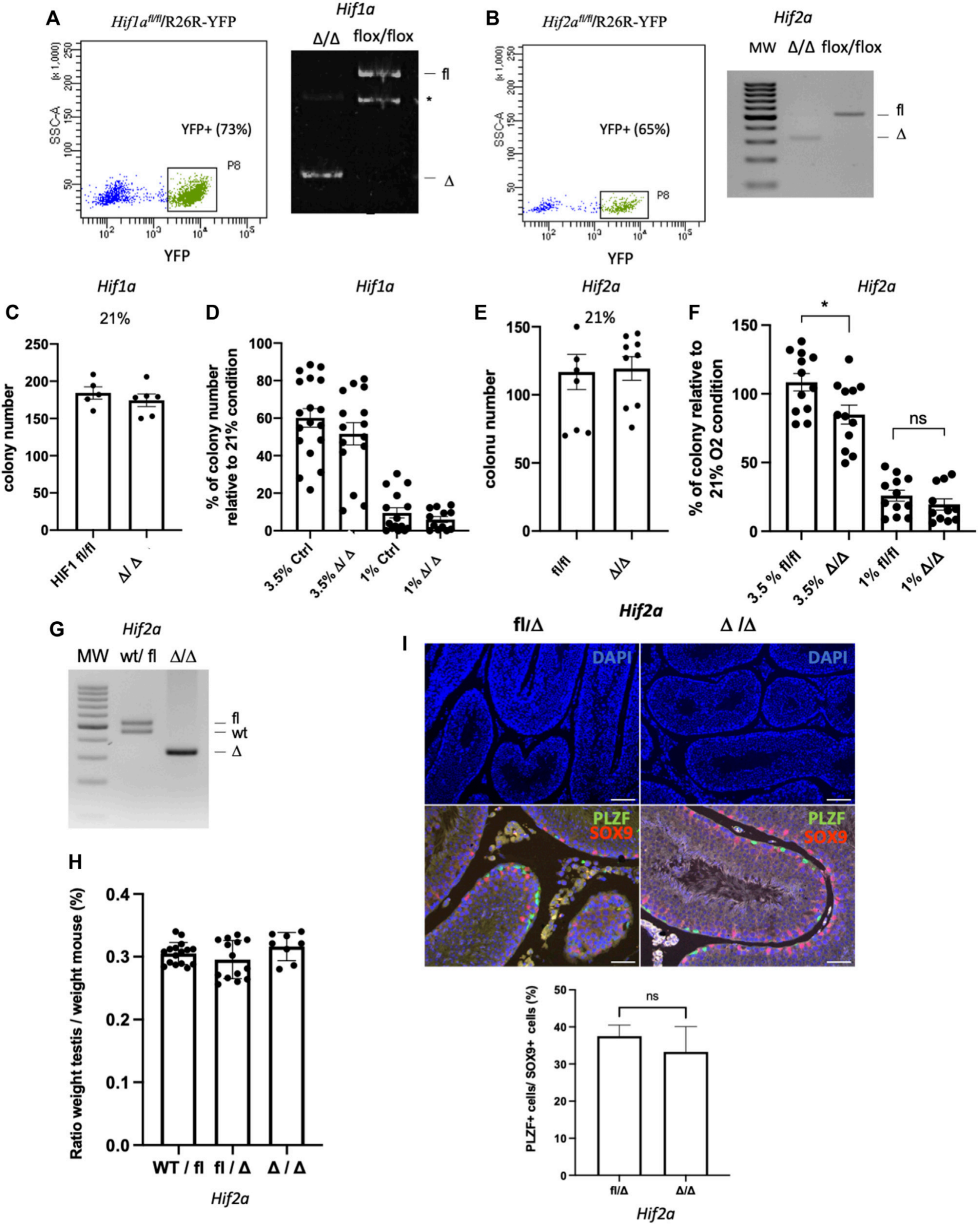
HIF-1α and HIF-2α in the response of SSCs to hypoxia **(A)** Flow sorting of YFP^+^SSCs 5 days after AAV-CRE transduction *in vitro* of *Hif1α* ^*fl/fl*^/R26R-YFP SSCs, and PCR genotyping of the resulting *Hif-1α* ^Δ/Δ^ SSCs showing excision of *Hif-1α* (*: unspecific band). **(B)** Flow sorting of YFP^+^SSCs 5 days after AAV-CRE transduction *in vitro* of *Hif2α* ^*fl/fl*^/R26R-YFP, and PCR genotyping of the resulting *Hif-2α* ^Δ/Δ^ SSCs showing excision of *Hif-2α*. **(C)** *In vitro* cell cluster forming ability of *Hif-1α* ^*Δ/Δ*^ SSCs in 21% O_2_ condition (*Hif-1α* ^*fl/fl*^ *n* = 5; *Hif-1α* ^*Δ/Δ*^, *n* = 6). **(D)** *In vitro* cell cluster forming ability of Ctrl and *HIF-1α* ^*Δ/Δ*^ SSCs in hypoxic 3.5% and 1% O_2_ conditions. *HIF-1α* ^*fl/fl*^ *,* ROSA-YFP+, and EGFP SSCs used as controls show similar *in vitro* cell cluster forming ability in 21% O_2_ condition (data not shown). **(E)** *In vitro* cell cluster forming ability of *Hif-2α* ^*Δ/Δ*^ SSCs in 21% O_2_ condition (*n* = 9). **(F)** *In vitro* cell cluster forming ability of *Hif-2α* ^*fl/fl*^ and *Hif-2α* ^*Δ/Δ*^ SSCs in hypoxic 3.5% and 1% O_2_ conditions (3.5% O_2_, *Hif-2α* ^*fl/fl*^ and *Hif-2α* ^*Δ/Δ*^, *n* = 12; 1% O_2_, *n* = 12 *Hif-2α* ^*fl/fl*^, *n* = 11 *Hif-2α* ^*Δ/Δ*^; 0.1% O_2_, *Hif-2α* ^*fl/fl*^ and *Hif-2α* ^*Δ/Δ*^, *n* = 9). **(G)** PCR genotyping showing excision of *Hif-2α* in germinal cells from testis of *Hif2* ^*fl/∆*^ *;Vasa-Cre* cKO mice. **(H)** Testis/body weight ratios of >7-month-old mice. No significant (ns) difference was observed in testis/body weight between *Hif2α* ^*wt/fl*^ (*n* = 16 testis, 8 mice), *Hif2α* ^*fl/Δ*^ (*n* = 14, 7 mice)*,* and cKO mice (*n* = 8, 4 mice). **(I)** Number of PLZF^+^ undifferentiated spermatogonia in *Hif2α* ^*fl/Δ*^ (*n* = 4)*,* and cKO (*n* = 3) mice (>7-month-old). Representative histological analysis of DAPI staining (DNA counterstained with DAPI, scale bar is 100 μm), and co-immunolabelling of PLZF undifferentiated spermatogonial marker (green) and SOX9 Sertoli cell marker (red) on testis sections, DNA is counterstained with DAPI (blue, scale bar is 50 μm). The quantification of the number PLZF^+^ undifferentiated spermatogonia is expressed as the ratio of PLZF^+^ cells per SOX9^+^ Sertoli cells.

HIF-1α and HIF-2α transcription factors are key modulators of the cellular response to hypoxia. HIF-2α mRNA was found enriched in murine β2M^−^α-6^+^c-kit^−^ undifferentiated spermatogonia in a transcriptome analysis that we previously performed (Givelet et al., 2022), and HIF-1α is expressed in spermatogonia (Takahashi et al., 2016). In addition, we observed that HIF-1α and HIF-2α are expressed in SSCs cultures under hypoxia (Supplementary Figure S1). We investigated the role of HIF-1α and HIF-2α transcription factors in SSCs. Using models of *Hif1α* (Ryan et al., 2000) and *Hif2α/Epas1* (Gruber et al., 2007) conditional allele mice that were crossed with R26R-YFP reporter mice, we established *Hif1α* ^*fl/fl*^/R26R-YFP *and Hif2α* ^*fl/fl*^/R26R-YFP SSCs culture. CRE excision of floxed DNA sequences were performed using AAV-CRE *in vitro*, and YFP-positive cells were sorted to create *Hif1α* ^*Δ/Δ*^ (Figure 7A) and *Hif2α* ^*Δ/Δ*^ SSCs (Figure 7B). We observed that *Hif1α* ^*Δ/Δ*^ SSCs did not show any alteration of their capacity to form germ cell clusters *in vitro* at 21% (Figure 7C), nor in hypoxic conditions at 3.5% or 1% O_2_ (Figure 7D). Hence, *Hif1α* ^*Δ/Δ*^ SSCs sound to behave normally in 21% O_2_ and hypoxic environment. Next, we tested the functionality of *Hif2α* ^*Δ/Δ*^ SSCs generated with the same protocol (Figure 7B). In 21% O_2_, *Hif2 α* ^*Δ/Δ*^ SSCs have the same colony-forming activity than control *Hif2α* ^*fl/fl*^ cells (Figure 7E). But, hypoxia at 3.5% sounds to affect slightly the activity of *Hif2α* ^*Δ/Δ*^ SSCs (Figure 7F), suggesting that HIF-2α, but not HIF-1α, could be involved in the response of SSCs to mild hypoxia *in vitro*. Postnatal *Hif2α* ablation in somatic and germ cells was previously reported to lead to male infertility, with reduced testis weight and a severe effect on spermatid and spermatozoa numbers but not affecting immature germ cells. However, the phenotype on the number of germinal cells worsened in old mice (>7 months) with seminiferous tubules depleted of germ cells, potentially suggesting an effect on spermatogonia with aging (Gruber et al., 2010). Hence, we generated *Hif2α* ^*fl/∆*^ *;Vasa-Cre* animals to specifically invalidate *Hif2α* in germ cells (Figure 7G) in order to analyse their testis in old males (>7 months). We did not observe any effects on the testis weight (Figure 7H). Histological analysis of cross-sections through control and *Hif2α*Δ/Δ samples revealed a proper architecture of the seminiferous tubules and the presence of PLZF^+^ undifferentiated spermatogonia and germ cells at the various differentiation stages (Figure 7I). We determined the frequency of the specific developmental subsets in the *Hif2α*Δ/Δ and control mice by flow cytometry using DNA content staining, α6-integrin and KIT markers in order to analyse spermatids, spermatocytes II and I, and differentiating and undifferentiated spermatogonial populations. The frequency of each stage of differentiation (premeiotic, spermatocytes I and II, spermatids) were equivalent (Supplementary Figure S2), showing no alteration or specific arrest of spermatogenesis. Specifically, no changes were observed in the frequency of spermatogonial progenitor populations, and notably in the α6^+^c-Kit^+^ differentiating spermatogonia and the α6^+^c-Kit^−^ undifferentiated spermatogonial populations, which contains SSCs. In addition, we counted the PLZF-positive undifferentiated spermatogonia in testicular sections of the *Hif2α*Δ/Δ and control mice. The number of PLZF-positive undifferentiated spermatogonia were similar in testis of the *Hif2α*Δ/Δ and control mice (Figure 7I), in agreement with the results obtained on the frequency of α6^+^c-Kit^−^ undifferentiated spermatogonial population. Altogether, those data suggest that *Hif2α* does not play a cell autonomous role in the regulation of the undifferentiated spermatogonial pool, containing SSCs.

## Discussion

In the testis, SSCs reside in a hypoxic environment, with oxygen concentrations as low as 1.5% detected within seminiferous tubules in the basement membrane (Gruber et al., 2010). “Physiologic normoxia” for SSCs appears to be at low levels of O_2_ tension and close to levels found in the bone marrow niche for hematopoietic stem cells, ranging from 1% to 4% (Nombela-Arrieta et Silberstein, 2014). In addition, we can assume that oxygen concentrations should decrease below 1% as cells progress in the spermatogenic process toward the lumen, supporting a role for hypoxia in the process of spermatogenesis. Hypoxia regulates the fate of fetal and adult stem cells from various lineages and is thought to be involved in the modulation of their quiescent state and level of proliferation through the lack of oxygen and the metabolic shift from mitochondrial oxidative phosphorylation toward anaerobic glycolysis (Nombela-Arrieta et Silberstein, 2014). Here, we studied the effects of low O_2_ tension (3.5%, 1%, and 0.1% O_2_) on the well-characterized murine SSCs in culture.

We observed a deleterious effect of severe hypoxia (1% O_2_ and 0.1% O_2_) on their capacity to form germ cell clusters when plated at low density. We could not culture adult SSCs at long term in high cell density conditions (30,0000 cells/0.32 cm^2^) under 3.5% O_2_ hypoxic environment. Clearly, the robust effect on the amplification of SSCs number observed in culture at high cell density under 21% O_2_ was impaired in hypoxic conditions. A negative effect of hypoxia on culture of neonatal SSCs from DBA/2 genetic background was recently reported in conditions close to our high cell density conditions (Morimoto et al., 2021). Here we investigated the effects of hypoxia on the regenerative potential of cultured SSCs, notably on adult SSCS from C57BL6/J background. It has been described that SSCs from DBA/2 and B6 backgrounds exhibit significant differences according to their regenerative potential after transplantation and their potential *in vitro* in SSCs culture, DBA/2 SSCs background promoting SSCs selfrenewal (Kanatsu-Shinohara et al., 2016). However, the effects of hypoxia on SSCs appeared similar in both genetic background in high cell density culture conditions. The cell density is a critical parameter for the success of SSCs culture at 3.5% O_2_ compared to 21% O_2_, and only cultures at low cell density were efficient. SSC cultures were performed on a feeder layer of MEFs. We observed an effect of hypoxia on the feeders and the negative outcomes on parameters such as pH and lactate levels in the medium, even if this feeder is inactivated by mitomycin C. Hypoxia could also induce the secretion of deleterious factors for SSCs or molecules promoting differentiation. Analysis of secretomes of the MEF feeders under the different hypoxic conditions could help to determine whether hypoxia indirectly influences, via the feeders, the fate of stem cells, their differentiation, their proliferative state and their colony-forming activity.

As O_2_ deprivation influences the rate of cell proliferation, the cell cycle status of SSCs was analyzed under the different culture conditions. A significant reduction of cells in S phase was observed when cultured under 1% O_2_ and 0.1% O_2_ conditions, preventing G_1_/S transit and progression to S phase. In contrast, proliferation rates were similar 12 h after seeding under 3.5% O_2_ and 21% O_2_ conditions. Thus, these proliferative defects could partly explain the impaired capacity of SSCs to form colonies. Of note, hypoxia moderately induced quiescence in SSCs, as shown by the 0.1% O_2_ culture. Recent studies showed that the culture of prepubertal and adult SSCs at 10% O_2_ apparently did not affect the proliferation rate of SSCs culture, in agreement with our data at 3.5% O_2_ (Helsel, Oatley, et Oatley, 2017), even if the regenerative capacity of SSCs in culture was improved at 10% O_2_. In contrast, 5% O_2_ hypoxia was reported to stimulate the proliferation of mouse germinal stem cells derived from neonatal 0-2-day-old mice (Kuo et al., 2018). Thus, severe effects on SSCs proliferation appear to occur at O_2_ tensions ≤1%. However, we showed in the present study that even if proliferation is drastically decreased in the same manner as SSCs functionality under severe hypoxia, the proliferation of germ cells in SSC culture is not completely blocked, and a fraction of PLZF^+^ cells (20%) still proliferate under 0.1% O_2_. The existence of two different discrete niches linked to the level of hypoxia due to their relative distance from blood vessels was recently suggested (Lord et Nixon, 2020). In the physiological context of the testis, our *in vitro* data concerning the effect of hypoxia on the proliferation status of SSCs could suggest that the regions close to vessels in interstitial tissue (higher O_2_ tension) and tubule-tubule/avascular interstitial regions (lower O_2_ tension) could correspond respectively to a proliferative niche (with an O_2_ tension >1%) and a niche less proficient at proliferation (O_2_ tension ≤1%). Niche oxygen levels influence both stem cell proliferation and differentiation. Hypoxia was shown to induce a slight bias toward differentiation under 1% and 0.1% O_2_ conditions.

The mitochondria play a major role into oxygen sensing pathways, through modulation of oxidative phosphorylation and ROS production. A positive effect of ROS generation on SSCs maintenance was recently described, as moderate ROS levels are tightly regulated in SSCs (Morimoto et al., 2013). The alteration of mitochondrial activity was recently reported in aged culture (60 months) of SSCs, with diminished ROS levels (Kanatsu-Shinohara et al., 2019). However later in the differentiation process, differentiating spermatogonia and meiotic spermatocytes need also mitochondrial fusion (Varuzhanyan et al., 2019). On the other hand, chemical inhibition of glycolysis using the glucose analogue 2-Deoxy-d-glucose impaired also SSC self-renewal capacity when grown at 21% O_2_ (Kanatsu-Shinohara et al., 2016). Altogether, SSCs sound to use glycolysis with a complex interplay with ROS, but progressively shift to oxidative metabolism when they differentiate. In line with this*, Slc2a1* (GLUT1) was recently described to be expressed in spermatogonia and progressively downregulated during meiotic initiation (Zhang et al., 2023). Cells in hypoxic environment were thought to adapt mitochondrial activity (Chandel et al., 1998). However, we did not observe significant changes of the mitochondrial mass and the mitochondrial potential of membrane in SSCs exposed to mild or severe hypoxia in our experimental conditions. Superoxide generated by mitochondria as a consequence of oxidative phosphorylation was observed to increase in 3.5 and 1% O_2_ conditions compared with 21% O_2_. On one hand, mitochondrial metabolism and OXPHOS seems to be activated in spermatogonia in 3.5% and 1% O_2_ hypoxic conditions. On the other hand, the increase of the lactate production in the medium of SSCs cultivated 5 days at 3.5 O_2_ could reflect a shift to glycolysis metabolism of SSCs in hypoxic condition. Increased glycolysis associated with the maintenance of the mitochondrial membrane potential might indicate compensatory mechanisms to respond to energy demand in hypoxic SSCs. Hence, a fine regulation of the equilibrium between OXPHOS and glycolytic metabolism must occur to maintain proliferation and renewal of SSCs in moderate hypoxia at 3.5% O_2_. In addition to mitochondria, the cytosolic NADPH oxidase can also be involved in hypoxia induced ROS production. Nox1 and *Nox3* genes were shown also to be involved in ROS generation in SSCs, revealing the complex regulation of this process (Morimoto et al., 2013; Morimoto, Kanatsu-Shinohara, et Shinohara, 2015; Morimoto et al., 2021). This origin of ROS production in hypoxia warrants further investigation in SSCs.

The molecular mechanisms explaining how hypoxia regulates SSCs remain largely unknown, even though glycolytic conditions of culture (Helsel, Oatley, et Oatley, 2017) and Myc-mediated glycolysis (Kanatsu-Shinohara et al., 2016) were reported to favor SSC self-renewal. Hypoxia induces a glycolytic program instead of mitochondrial oxidative phosphorylation. HIFs are key modulators of the cellular response to hypoxia. Under 21% O_2_ conditions, the alpha subunit (HIF-1α or HIF-2α) is hydroxylated by prolyl hydroxylase (PHD) enzymes and is directed to the proteasome for degradation after HIF1α ubiquitination mediated by the Von Hippel Lindau (VHL) tumor suppressor factor. In hypoxia, HIF-1α protein accumulates due to PHD enzyme inactivity. Then, HIF-1α translocates into the nucleus and binds to the beta subunit (HIF-1β), inducing the expression of target genes in the low O_2_ tension environment. HIF-2α is the other partner of HIF-1β. HIFs are essential for early mammalian development and stem cell maintenance of various tissues (Keith et Simon, 2007). Our data tend to show that HIF-1α is not involved in the SSC response to hypoxia whatever the level of hypoxia. These data are in agreement with the apparently normal phenotype of spermatogenesis previously reported for HIF-1α mice conditionally inactivated in germinal and somatic testicular cells (Gruber et al., 2010). However, HIF-1α was recently identified as a signaling pathway that potentially regulate SSCs in adult, in a study searching for signaling pathways encompassing paracrine factors from the niche and membrane proteins expressed on SSCs in mice and human (Jin et al., 2023). Another recent study showed the role of HIF-1α in the regenerative capacity of SSCs following transplantation (Morimoto et al., 2021), despite the lack of effects on spermatogenesis of a conditional inactivation of HIF-1α in testicular cells (Gruber et al., 2010). Altogether, these data suggest a complex role of HIF-1α in SSCs renewal likely depending on the homeostatic and regenerative tissue status, but also that could be independent of hypoxia. HIF-2α is required for the maintenance of PGCs in embryos (Covello et al., 2006) and regulates the *Oct4* target gene, which is also important for the maintenance of undifferentiated spermatogonia. Recently, HIF-2α was reported to be involved in the response of neonatal germline stem cells to hypoxic conditions and *Oct4* regulation (Kuo et al., 2018). Our data showing a slight decrease of the colony-forming-activity of SSCs suggest that HIF-2α could be implicated in the maintenance of SSCs at 3.5% O_2_, but not in more severe hypoxia. Postnatal HIF-2α ablation at P2, using a conditional model of invalidation, leads to reduced testis size and a loss of germinal cells, with the phenotype worsening with age (Gruber et al., 2010), suggesting a role in spermatogenesis. But these observations did not allow to determine whether this spermatogenetic impairment was linked to somatic and/or germinal defects. Here, we show that HIF-2α *in vivo* does not play a cell autonomous role in SSCs and germinal cells. Apart from HIFs, hypoxia is known to activate also pathways like the mTOR complex 1 (mTORC1), the Unfolded Protein Response pathways, and other transcription factors such as the NF-κB transcription factor family and its interplay with ROS (Morgan et Liu, 2011; Lee, Chandel, et Simon, 2020). The expression of NF-κB was described in spermatogonia but its role is barely known (Wright et al., 2007). Interestingly, JNK hyperactivation and enhanced glycolysis was reported in aging of SSCs (Kanatsu-Shinohara et al., 2019), and NF-κB exerts its anti-apoptotic function by the downregulation of the JNK pathway (Morgan et Liu, 2011). In addition, it was recently reported that the loss of HIF signaling in leukemic and normal hematopoietic stem cells did not alter the hypoxia-induced glycolytic state, suggesting an HIF independent response to hypoxia that could be mediated by the BCR-ABL oncogene (Wierenga et al., 2019). The role of these HIF independent pathways in the response of SSCs to hypoxia should be evaluated in future studies.

The amplification of SSCs *in vitro* constitutes an important step in order to increase the stem cell number obtained from testicular biopsies, as well as in the future use of SSCs in cell therapy. Even if SSCs reside in a hypoxic environment in the testis, our data show that the cell autonomous response of SSCs *in vitro* to hypoxia does not sound to be as critical as expected. Human SSCs still cannot be amplified at long term *in vitro* (Wu et al., 2009; Medrano et al., 2016). Despite the previously reported increase in the efficiency of establishing murine primary cultures under glycolytic-optimized conditions (Helsel, Oatley, et Oatley, 2017), human SSCs were only maintained *in vitro* up to 2–3 weeks when cultured under 3.5% O_2_ hypoxia and did not show any improvement compared with the results under 21% O_2_. Similarly, the supplementation with small molecules favoring the glycolytic pathway in mouse SSCs (Kanatsu-Shinohara et al., 2016) had no effects on the maintenance of human SSCs (data not shown). In our conditions, hypoxia appears to not be a critical key factor in the propagation of human SSCs. 3-dimensional (3-D) culture systems could constitute very interesting alternatives to propagate human SSCs through the use of human tissue decellularized extracellular matrix scaffolds, of 3D-bioprinting techniques, and the association of SSCs with human somatic testicular cells from the niche or with pluripotent stem cell-derived cells recapitulating the testicular niche (Lara, Sakib, et Dobrinski, 2021; Murdock et al., 2019). Future works should be done in order to identify key parameters in order to recapitulate artificially *in vitro* the niche enabling the amplification of SSCs in culture.

## Materials and methods

### Experimental model and human material

The C57BL/6, *Hif1α* ^*fl/fl*^ (B6.129-Hif1α^tm3Rsjo^/J strain from The Jackson Laboratory), *Hif2α* ^*fl/fl*^ (Gruber et al., 2007), Vasa-cre (Gallardo et al., 2007) and R26R-YFP mice were housed in our animal facility. Experiments were performed in compliance with European legislation and the Ethics Committee of the French Ministry of Agriculture (Agreement B9203202). Generation and genotyping methods of *Hif2α* ^*fl/fl*^ and *Hif1α* ^*fl/fl*^ mice have been previously described (Gruber et al., 2007; Toullec et al., 2018). *Hif1α* ^*fl/fl*^/R26R-YFP *and Hif2α* ^*fl/fl*^/R26R-YFP mice were obtained by crossing with R26R-YFP mice. Germ cell specific inactivation of *Hif2α* was achieved by cross-breeding *Vasa-Cre* transgenic mice (The Jackson Laboratory) with *Hif2α* ^*fl/fl*^/mice. *Hif2α* ^*fFl/∆*^ *; Vasa-Cre* animals were generated. Cre-mediated recombination between the loxP sites in the 2-loxP allele produces the 1-loxP allele, which lacks exon 2 and produces a mutant mRNA transcript containing multiple in-frame stop codons downstream of exon 1 sequences. The WT, 2-loxP, and 1-loxP allele can be distinguished by a multiplex PCR [primer 1: 5-CAGGCAG TAT​GCC​TGG​CTA​ATT​CCA​GTT-3; P2: 5-CTTCTTCCATCAT​CTG​GGA​TCT​GGG​ACT-3; P3: 5-GCTAACACTGTAC​TGT​CTG​AAA​GAGTAG​C-3]. The WT allele produces a 450-bp fragment (P1 and P2), the 2-loxP allele produces a 530-bp fragment (P1 and P2), and the 1-loxP allele produces a 360-bp fragment (P1 and P3). For *Hif1α*, primers GGT​GCT​GGT​GTC​CAA​AAT​GT (forward) and GGG​CAG​TAC​TGG​AAA​GAT​GG (reverse) were used to detect a 345-bp product for the floxed HIF-1α allele and a 295-bp product for the wild-type allele, Adult human testis biopsies were obtained via the CECOS Hospital Cochin from obstructive azoospermia patients, showing normal spermatogenesis, who consented to the study (IRB approved protocol: IRB 00003835; 2012/40ICB; French Institutional Review Board-Comité de Protection des Personnes, Ile de France IV).

### Testicular single-cell suspensions and immunomagnetic and flow cell sorting

Murine testicular single-cell suspensions were prepared from pups or 2-3-month-old C57BL/6 mice as previously described (Lassalle et al., 2004). The immunomagnetic selection of α6^+^ cells was performed using anti-α6 integrin-PE (GoH3) (BD Pharmingen) and anti-PE microbeads (Miltenyi Biotec) according to the manufacturer’s protocol. Hoechst staining (5 μg/mL) of the cell suspensions was performed as described previously (Lassalle et al., 2004). Cells were labeled with β2M-FITC (Santa Cruz), anti-EpCAM APC (Biolegend) and anti-CD117-APC (2B8) antibodies (BD Pharmingen). Propidium iodide (Sigma) was added before cell sorting to exclude dead cells. Cell sorting was performed on ARIA flow cytometers (Becton Dickinson).

Cells were isolated from azoospermic men with normal spermatogenesis. The seminiferous tubules were dissociated using enzymatic digestion by collagenase type I at 100 U/ml for 25 min at 32°C in 0.25% trypsin. After an HBSS wash, the resulting whole cell suspension was successively filtered through a 40-μm nylon mesh to remove cell clumps. After an HBSS wash, the cell pellet was resuspended in incubation buffer (HBSS supplemented with 20 mM HEPES pH 7.2, 1.2 mM MgSO_4_7H_2_O, 1.3 mM CaCl_2_2H_2_O, 6.6 mM sodium pyruvate, 0.05% lactate, glutamine and 1% fetal calf serum) and further incubated at 32°C in a water bath. Cell concentrations were estimated using Trypan Blue staining (>95% viable cells). Cells were then labeled with anti-α6 integrin-PE (GoH3), β2m-FITC, and anti-THY1-APC antibodies (BD Pharmingen).

GFRA1 was labelled using goat polyclonal anti-GFRA1 (R&D, AF560) and anti-goat-BV421 (Jackson immunoresearch) secondary antibodies. Propidium iodide (Sigma) was added before cell sorting to exclude dead cells. Cell sorting was performed on ARIA flow cytometers (Becton Dickinson). Cell death in cultured was measured using Hoechst 33258 to stain dead cells. Analyses were performed on LSR II flow cytometers (Becton Dickinson).

### SSCs and MEF culture

To derive adult SSC lines from adult C57BL/6, *Hif1α* ^*fl/fl*^/R26R-YFP *and Hif2α* ^*fl/fl*^/R26R-YFP SSCs mice, α6^+^c-Kit^−^/β2M^−^ germinal cells were isolated and maintained on mitomycin-C-treated MEFs as previously described (Barroca et al., 2009). The SSC culture medium was composed of Stem Span (Stemcell Technologies) and B27 supplement (Life Technologies) and supplemented with recombinant human GDNF (40 ng/mL, R&D Systems), recombinant rat GFRα1 (300 ng/mL, R&D Systems), FGF2 (1 ng/mL, Life Technologies), and ES-Cult™ Fetal Bovine Serum (1%, Stemcell Technologies). Every 3–4 days, SSC clusters were split using enzymatic digestion with 0.05% trypsin-EDTA (Life Technologies). SSCs were used for experiments after at least 1.5 months of culture. MEF cultures were established by trypsin digestion of C57BL/6 13.5 dpc embryos, and the resulting cells were cultured in DMEM supplemented with 10% FBS, L-glutamine and penicillin/streptomycin. SSCs were incubated at 37°C, 5% CO_2_ at various O_2_ tensions: 0.1%, 1%, 3.5% O_2_ and 21% O_2_. ProOx C21 carbon dioxide and oxygen controller and a C-Chamber (Biospherix, Redfield, NY, United States) were used to maintain the hypoxic 0.1%, 1% or 3.5% O_2_ conditions. *Hif1α* ^*Δ/Δ*^ and *Hif2α* ^*Δ/Δ*^ SSCs were generated by transduction of *Hif1a* ^*fl/fl*^/R26R-YFP *and Hif2α* ^*fl/fl*^/R26R-YFP SSCs in culture using adenovirus AAV-DJ expressing Cre recombinase. YFP-positive cells were sorted 6 days after transduction, and excision was controlled by PCR in *Hif1α* ^*Δ/Δ*^ and *Hif2α* ^*Δ/Δ*^ SSCs generated. The pH, pCO_2_, pO_2_ and lactate concentration in medium were measured using an ABL800 blood gas analyser (Radiometer, Copenhagen, Denmark).

Human α6^+^THY1^+^β2M^−^ germinal cells were isolated and maintained on mitomycin-C-treated MEFs as previously described (Barroca et al., 2009) or on gelatin. The SSC culture medium was composed of Stem Span (Stemcell Technologies) and B27 supplement (Life Technologies) and supplemented with recombinant human GDNF (40 ng/mL, R&D Systems), recombinant rat GFRα1 (300 ng/mL, R&D Systems), FGF2 (1 ng/mL, Life Technologies), and ES-Cult™ Fetal Bovine Serum (1%, Stemcell Technologies).

### Immunofluorescence

Testis were fixed in 10% neutral-buffered formalin overnight at 4°C, dehydrated, embedded in paraffin wax and cut into 5 μm thick sections. Sections were mounted on slides and dewaxed. For immunofluorescence, rehydrated sections were submitted to antigen retrieval by boiling for 20 min in citrate buffer (pH 6). After cooling, sections were permeabilized by incubating the slides in PBS1X-Triton 0.2% for 10 min and then blocked for 30 min in PBS with 5% bovine serum albumin (BSA). Slides were rinsed in PBS1X before incubation 1 h at RT with the primary antibodies diluted in PBS1X-BSA 0.5% blocking buffer: anti-PLZF goat polyclonal antibody (1/200e, R&D, AF2944) and anti-SOX9 rabbit monoclonal antibody (1/200e, abcam, ab185230). After rinsing several times, slides were incubated for 1 h with the appropriate secondary antibodies and 4,6-diamidino-2-phenylindole (DAPI, 0.4 mg/mL) for DNA staining: donkey anti-goat Alexa 488 antibody (1/200e, Invitrogen, A11055) and donkey anti-rabbit Alexa 594 antibody (1/200e, Invitrogen, A21207). Finally, slides were mounted with coverslips in fluorescence mounting medium (S302380, Dako or ProLong Gold, P10144, Life Technologies). For HIF-1α and HIF-2α immunolabelling, wild type SSCs in culture were fixed with PFA 4% and permeabilized with Triton X-100 0.2% HIF-1α and HIF-2α were respectively detected using rabbit anti-HIF-1α (NB100-479, Novus) and rabbit anti-HIF-2α (NB100-122, Novus) and donkey anti-rabbit Alexa 488 antibody (1/200e, A212006, Invitrogen). Controls were labelled with rabbit (DA1E) IgG rabbit and donkey anti-rabbit Alexa 488 antibody (1/200e, A212006, Invitrogen). Imaging was performed using a confocal NIKON A1 microscope or an Olympus AX70 epifluorescence microscope equipped with a CoolSNAP Myo camera Photometrics and Micro-Manager software (version 1.4.16, open source microscopy).

### Testicular transplantation

C57BL6 mice were used as the recipients for cell transplantation. To deplete endogenous spermatogenesis, the recipient mice were treated at 6–8 weeks of age with busulfan (40 mg/kg at least 4 weeks before donor cell transplantation). Donor cells were resuspended in DMEM supplemented with 10% heat-inactivated fetal calf serum, 100 mg/mL DNase I (DN25, Sigma-Aldrich) and 4% trypan blue solution (T8154, Sigma-Aldrich) for transplantation as previously described (Barroca et al., 2009). A 10-microliter solution of donor cells was introduced into the seminiferous tubules of the testis of the recipient mouse via an injection through the efferent ductules as previously described. Ten weeks after transplantation, the recipient testis were collected and analyzed by either macroscopic observation of fluorescence in mice. Macroscopic observations of recipient testis tissues were performed using an Olympus epifluorescence microscope to detect the presence of EGFP- fluorescent seminiferous tubules.

### BrdU experiment

First, 100,000 cells were inoculated in 24-wells plates with a volume of 500 µL. The cells were incubated at 37°C for 24 h under different conditions of hypoxia (21%, 3.5%, 1% and 0.1%). BrdU at the final concentration of 30 µg/L was added before the termination of culture. Then, 2 h later, the cells were fixed and permeabilized using a Cytofix/Cytoperm kit (BD Pharmingen). For the pulse-chase, 100,000 cells were inoculated in 24-wells plates with a volume of 500 µL. The cells were incubated at 37°C for 24 h under 21% O_2_. BrdU at a final concentration of 30 µg/L was added, and, 2 h later, the cells were rinsed and the solution was replaced. The cells were incubated at 37°C for 24, 34, 48, 58, or 72 h under different conditions of hypoxia (21%, 3.5%, 1% and 0.1%). The cells were fixed and permeabilized using a Cytofix/Cytoperm kit (BD Pharmingen) according to the manufacturer’s protocol and stained with the following antibodies: FITC-anti-BrdU antibody (BD), and APC-anti PLZF antibody (R&D). Cell death was measured using Hoechst 33258 to stain dead cells. Analyses were performed on LSR II and FACSCalibur flow cytometers (Becton Dickinson).

### KI67 labeling

First, 100 000 cells were seeded in 24-wells plates with a volume of 500 µL. The cells were incubated at 37°C for 4 days under different condition of hypoxia (21%, 3.5%, 1% and 0.1%). The cells were fixed and permeabilized using a Cytofix/Cytoperm kit (BD Pharmingen) and stained with the following antibodies: FITC-antiKI67 antibody (BD) and APC-antiPLZF antibody (R&D). Analyses were performed on LSR II and FACSCalibur flow cytometers (Becton Dickinson).

### Analysis of mitochondria

After 24 h in 21% O_2_/hypoxic conditions on attachment factor-coated 24-wells plate, 10^5^cells in SSCs medium loaded with 5 μM mitoSOX Red (M36008; Molecular Probes) were incubated in the dark for 30 min at 37°C in 21% O_2_ and hypoxic conditions. For the mitochondrial mass and the mitochondrial membrane potential, cells were loaded with 20 nM MitoTracker Green (M7514; Molecular Probes) and TMRE (100 nM) in the dark for 30 min at 37°C. Cells were incubated for 20 min at 37°C with 50 mM FCCP (carbonyl cyanide 4-(trifluoromethoxy)phenylhydrazone) to test the specificity of TMRE. The fluorescence was measured using LSRII flow cytometer (Becton Dickinson).

### Statistical analysis

Statistical analysis was performed in Graphpad Prism 9. Results are represented as mean ± SEM. When comparing more than 2 groups, ANOVA was performed with *post hoc* Tukey correction for multiple comparisons. When only 2 groups were compared, an unpaired Mann whitney was used, unless otherwise stated. Statistical significance is indicated in Figures as follows: ^∗^ *p* < 0.05; ^∗∗^ *p* < 0.01; ^∗∗∗^ *p* < 0.001; ^∗∗∗∗^ *p* < 0.0001 unless otherwise stated.

## Data Availability

The original contributions presented in the study are included in the article/Supplementary Material, further inquiries can be directed to the corresponding author.
