# Supplementary figures and images for: Impact of the hypoxic microenvironment on spermatogonial stem cells in culture
Source: Front Cell Dev Biol. 2024 Jan 18;11:1293068. doi: 10.3389/fcell.2023.1293068 (PMC10830753; doi:10.3389/fcell.2023.1293068)

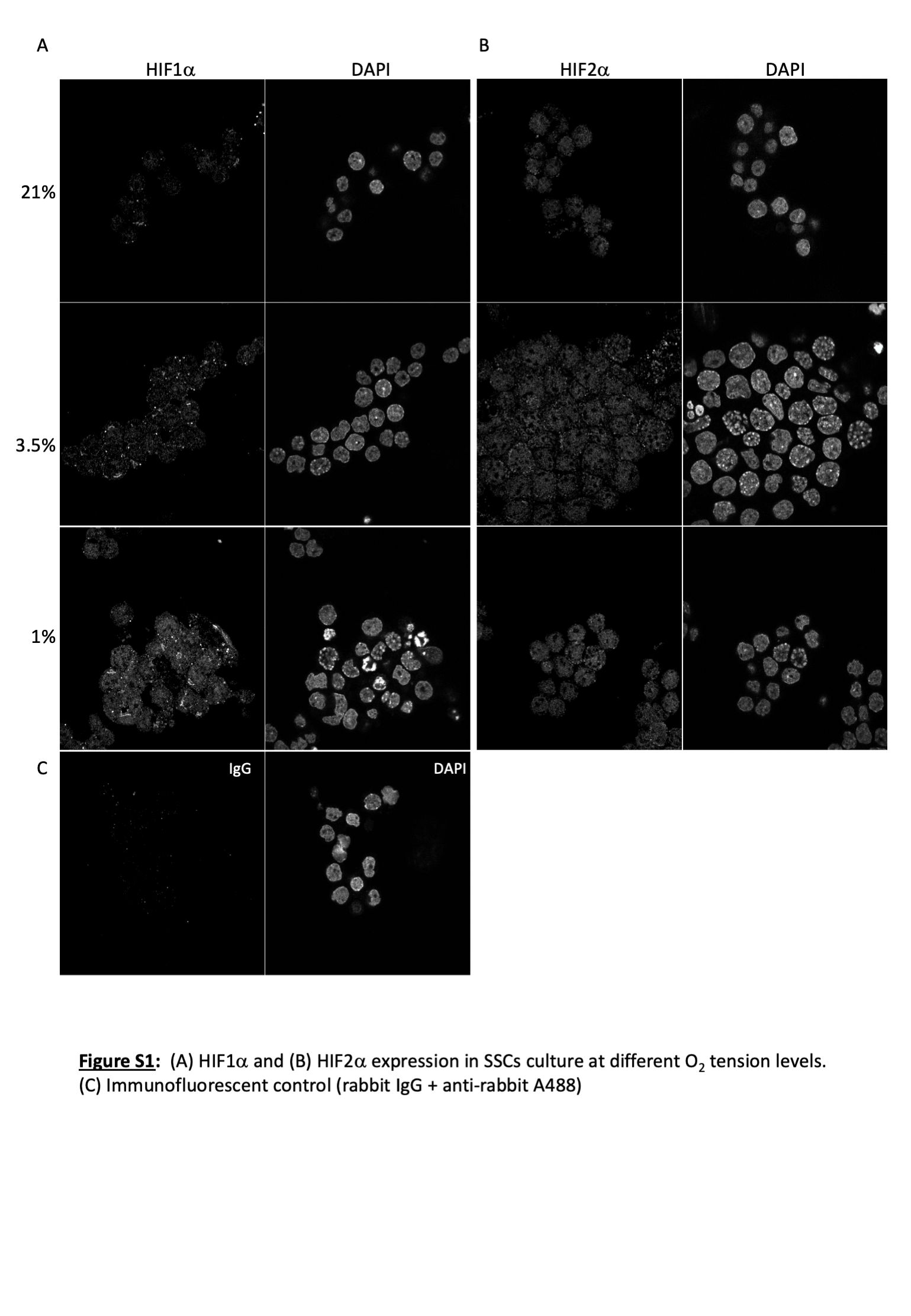

Supplement: Supplementary file 1 [file Image1.tiff]

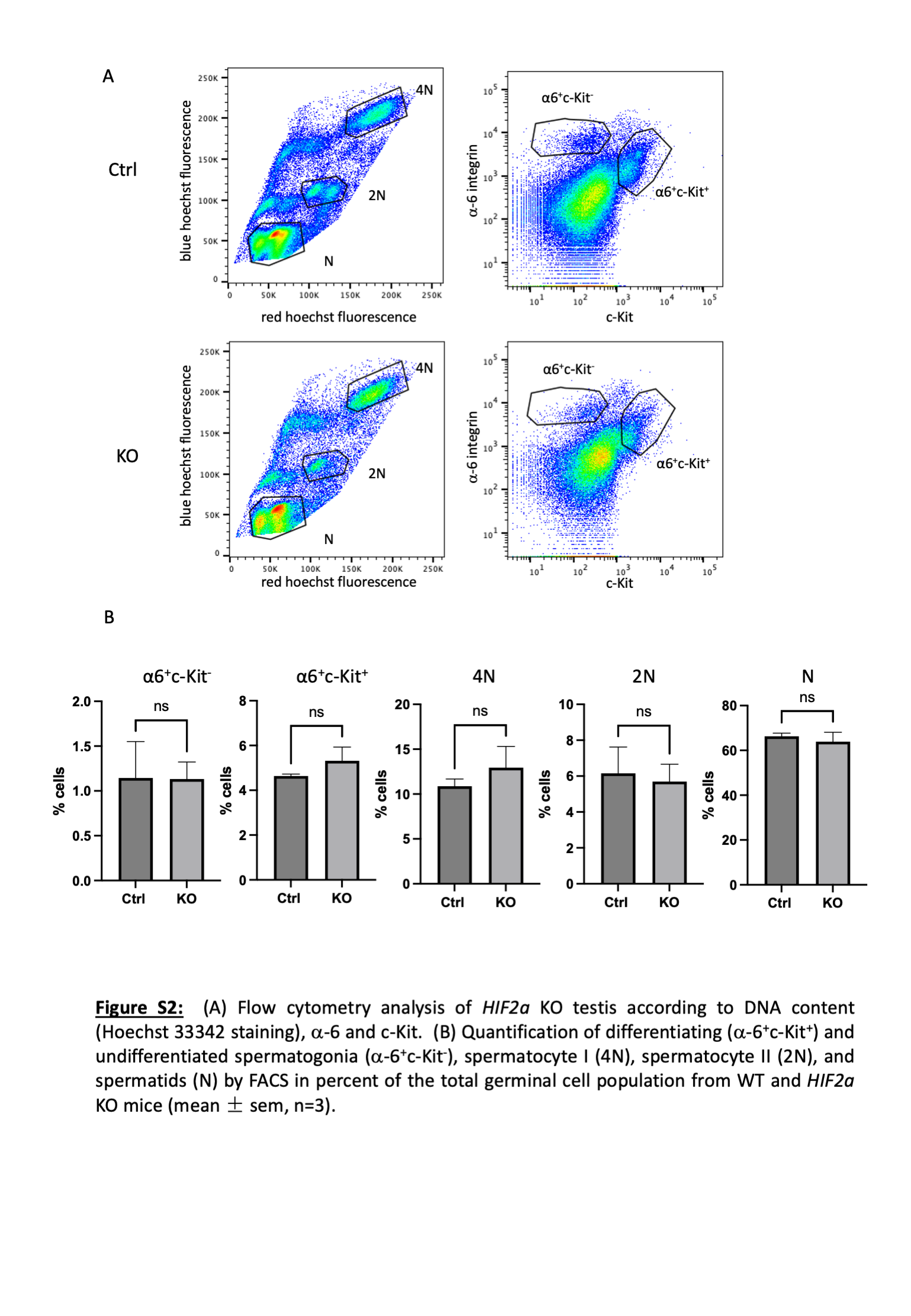

Supplement: Supplementary file 2 [file Image2.tiff]
